# Editorial: New professionalism and the future of work: interdisciplinary perspectives on transformations in business-health relationships, volume II

**DOI:** 10.3389/fpsyg.2025.1610961

**Published:** 2025-05-09

**Authors:** Gabriele Giorgi, Antonio Ariza-Montes, Nicola Mucci, Annamaria Di Fabio

**Affiliations:** ^1^Department of Health and Life Sciences, European University of Rome, Rome, Italy; ^2^Social Matters Research Group, Universidad Loyola Andalucía, Seville, Spain; ^3^Department of Experimental and Clinical Medicine, University of Florence, Florence, Italy; ^4^Specialist Occupational Medicine Unit, Careggi University Hospital, Florence, Italy; ^5^Department of Education, Languages, Intercultures, Literatures and Psychology (Psychology Section), University of Florence, Florence, Italy

**Keywords:** professional competencies (PCs), future of work (FoW), vocational training, workplace wellbeing, organizational culture (OC), digital transformation (DT), mental health, occupational health

## Introduction

This Research Topic represents a second volume of the Research Topic “*New Professionalism and the Future of Work: Interdisciplinary Perspectives on Transformations in Business-Health Relationships*” (Giorgi et al., 2019).

Around the world, the nature and meaning of work are rapidly evolving with fast and profound changes. These changes are driven by pressing innovations, technological and communication transformations, and social changes and these transformations are expected to shape the future of work. The concept of the classic workplace is also deeply changing, due to new emerging professions connected to digital work and technology leading to the development of new skills, the need of upskilling and reskilling and of a continuing professional development (Li, 2024; Le et al., 2024; Fan and Moen, 2023).

The seven manuscripts published in this Research Topic are both empirical contributions and reviews, involving multiple work professions. The manuscripts, when considered together, bring out two relevant aspects. First, professional competencies have a significant impact on workers' performance and well-being. Given that both new skills are required, a new professionalism appears fundamental. Second, vocational training seems to be crucial. Training programs should be provided across different organizational settings and professions targeting both the new generation and elderly workers.

## Overview of Articles in this Research Topic

The research findings emphasize the necessity of interdisciplinary approaches and evidence-based strategies to optimize business excellence and psychological wellbeing in workplace. Particularly, the development of professional competencies, the value of workplace mental health, the impact of occupational conditions on suicide prevention, the importance of organizational culture and the management of organizational identification highlight the imperative for healthy, sustainable, and human-centered work models for the future of work (Di Fabio and Cooper, 2023).

The study conducted by Ježková Petrů and Zychová looks into the vital skills needed for social service managers and what shapes their growth. By surveying various social service organizations in the Czech Republic, the study puts in light two main areas of competency: socio-legal counseling, along with analytical and conceptual skills, and the realms of diagnostics and social prevention.

The paper by Zientz et al. evaluates how a structured brain health training program affects workplace wellbeing and burnout levels. Over a six-month period, 193 employees participated in brief cognitive strategies and personalized coaching. After the program, 75% of participants reported improvements in their brain health, with those more engaged in the training experiencing better emotional balance and less job-related exhaustion.

As reported by Llosa et al., suicide appears as a pressing public health issue, pointing out its complex nature and the absence of universally effective prevention methods. Drawing on Abrutyn's psychosocial and holistic model, this research brings together recent empirical findings, stressing the need to incorporate occupational factors into suicide prevention strategies.

The study conducted by Hu investigates how work-family culture relates to job satisfaction within Chinese higher education institutions, focusing on the mediating role of organizational justice. Data from 1,075 faculty members and 972 administrative employees reveal that work-family culture significantly influences perceptions of organizational justice, which, in turn, mediates its effect on job satisfaction. These findings highlight the critical role of work-family culture.

The study of Wang et al. dive into how the psychological age climate influences the motivation of older workers in China. By analyzing data from 1,094 employees aged 50 to 70, the findings show that a supportive age climate boosts motivation, mainly through job autonomy and social support. Interestingly, factors like job design tailored to aged workers' skills and ergonomic working conditions didn't have a significant impact.

Abebe Boe et al., evaluate the level of professionalism among 405 nurses in Hawassa, Ethiopia. It reveals a moderate level of professionalism, shaped by factors such as education, professional memberships, work experience, and advanced qualifications. The findings point to a clear need for focused interventions to enhance professional standards and ongoing training programs in nursing.

The paper by Rovetta et al., through a review, discuss how team and organizational identification affects wellbeing, behavior, and effectiveness, using Social Identity Theory as a framework. While strong identification can boost employee wellbeing, over-identification with a specific team, as opposed to the organization as a whole, can lead to dysfunctional dynamics and reduce organizational effectiveness.

## Conclusion

As encapsulated in the guiding principle of the *Business@Health Laboratory* at the European University of Rome and at the *Work and Organizational Psychology for Healthy Organizations Laboratory* at the University of Florence:

“Business cannot exist without workers' health, and workers' health is business.”

This Research Topic of research provides valuable insights to guide organizations and policymakers in shaping a productive, healthy, and human-centered future of work with a peculiar focus on continuing professional development.

## References

[B1] Di Fabio A. Cooper C. L. (eds.). (2023). Psychology of Sustainability and Sustainable Development in Organizations. Routledge Taylor & Francis Group. 10.4324/9781003212157 PMC5610694 28974935

[B2] Fan W. Moen P. (2023). The future(s) of work? Disparities around changing job conditions when remote/hybrid or returning to working at work. Work Occup. 52, 91–129. 10.1177/07308884231203668

[B3] Giorgi G. Mucci N. Di Fabio A. Ariza-Montes A. (2019) Editorial: New professionalism the future of work: interdisciplinary perspectives on transformations in business-health relationships. Front. Psychol. 10:2193. 10.3389/fpsyg.2019.02193 31607999 PMC6769423

[B4] Le K. B. Q. Sajtos L. Kunz W. H. Fernandez K. V. (2024). The future of work: understanding the effectiveness of collaboration between human and digital employees in service. J. Serv. Res. 28, 186–205. 10.1177/10946705241229419

[B5] Li L. (2024). Reskilling and upskilling the future-ready workforce for industry 4.0 and beyond. Inform. Syst. Front. 26, 1697–1712. 10.1007/s10796-022-10308-y 35855776 PMC9278314

